# Mortality in Amputees with Peripheral Artery Disease during the Post-COVID Era: A Three-Year Analysis

**DOI:** 10.3390/diseases12070133

**Published:** 2024-06-27

**Authors:** Mohammad Mahdi Kasiri, Martina Mittlboeck, Bernd Gollackner, Christoph Neumayer

**Affiliations:** 1 Department of General Surgery, Division of Vascular Surgery, Medical University of Vienna, 1090 Vienna, Austria; 2 Institute of Clinical Biometrics, Center for Medical Data Science, Medical University of Vienna, 1090 Vienna, Austria

**Keywords:** COVID-19, mortality, pandemic, peripheral artery disease, vascular disorders, amputation, survival

## Abstract

Background: Patients with peripheral artery disease (PAD) have 40–70% higher three-year mortality after lower limb amputation compared to non-amputees. In this study, we examined the consequences of delayed treatment for patients with PAD during the coronavirus disease 2019 (COVID-19) pandemic. Methods: This study employed a retrospective single-centre cohort design at a large tertiary care hospital. We compared amputees with PAD during the initial COVID-19 outbreak period in 2020 with a control group of amputees from 2019 after a three-year follow-up. Results: In total, 134 amputees with PAD were included due to unsuccessful revascularization (*n* = 84 in 2020 vs. *n* = 50 in 2019). Patients in 2020 were significantly younger than those in 2019 (*p* = 0.01) and mostly admitted with advanced stages of PAD (*p* < 0.03). The proportion of major limb amputations increased significantly in 2020 (*p* = 0.03). Non-COVID-19-related deaths among patients in 2020 were more than twice as many as those in 2019, and long-term mortality in 2020 was 49% compared to 39% in 2019 (*p* = 0.04). Diabetes and renal insufficiency had a significantly negative impact on the survival of amputees with PAD (*p* < 0.01). Conclusions: Delayed treatment in patients with PAD leads to high long-term mortality risk after amputation, especially in PAD patients with diabetes and renal insufficiency. Therefore, in future pandemics, continuously monitoring patients with PAD will be crucial to prevent delayed treatment and severe short-term and long-term consequences.

## 1. Introduction

Peripheral artery disease (PAD) is a manifestation of systemic atherosclerosis and is associated with increased cardiovascular mortality. Patients with PAD are at a six-fold increased risk of mortality from cardiovascular causes compared to patients without PAD [1].

Despite advances in PAD care, including conservative, interventional, and surgical treatments, amputations are still occasionally required for the management of end-stage lower limb disease. Patients with PAD with lower limb amputation have 40–70% higher mortality during the first three years of follow-up compared to patients with PAD without amputation [2,3,4].

Recent findings suggest that during the coronavirus disease 2019 (COVID-19) pandemic, patients with chronic conditions such as PAD did not receive adequate healthcare services due to the disproportionate prioritization of COVID-19-related conditions [5]. This led to a dramatic increase in amputation rates reports dealing with lower extremity [6]. Despite growing knowledge about the management of COVID-19 infection in patients with PAD [7], limited data exist concerning the hidden effect of the COVID-19 pandemic on the clinical condition of non-COVID-19-infected patients with PAD, especially those with major limb amputation.

Therefore, as the international prevalence of PAD continues to rise [8,9], we aimed to analyse the short- and mid-term consequences of delayed treatment on the life expectancy of patients with PAD who have undergone amputation during the COVID-19 pandemic.

## 2. Materials and Methods

This study employed a retrospective, single-centre, observational cohort design from a prospectively maintained database at a large tertiary care hospital in Austria.

Inclusion criteria: we investigated clinical data from all patients with PAD who underwent amputation after an unsuccessful revascularization attempt due to acute or chronic situations and critical limb ischaemia during the COVID-19 outbreak period from March 2020 to December 2020. Exclusion criteria: all patients with a diagnosis other than PAD without amputation.

The patients have been invited for regular outpatient follow-ups since then. The control group consisted of amputees from the corresponding period in 2019. A total of 134 patients were included in this study: 84 from 2020, compared to 50 patients from the same period in 2019. This study was conducted over a three-year follow-up period and was registered with the ClinicalTrials.gov identifier NCT04838093. Patients’ clinical status at the time of amputation was assessed using the functional classification system of Rutherford [10]. Patients were assigned to two groups: 1) Rutherford classification 1–3 and 2) Rutherford classification 4–6. The group with Rutherford classification 1–3 contains only patients with PAD and stage 3 Rutherford. The group with Rutherford classification 4–6 contains only patients with PAD and stages 4, 5, and 6 Rutherford. Amputations performed at the level of the ankle joint or below were classified as minor amputations, while amputations performed above the ankle joint were classified as major amputations. We defined a blood creatinine level > 1.7 together with GFR < 89 mL/min as renal insufficiency. We defined a blood HbA1C level > 6.5 as diabetes.

This study was approved by the ethics committee of the Medical University of Vienna (EK 2049/2020) and conducted according to the principles of the Declaration of Helsinki and Good Clinical Practice protocols. Due to the retrospective nature of the study, informed consent was not required.

### Statistics

Baseline characteristics and outcome measures were summarized using frequencies and percentages. The distributions of continuous variables are shown by boxplots and described by the median, minimum, and maximum. To reveal differences between the two groups, Pearson’s χ2 test or Fisher’s exact test (in the case of sparse data for categorical variables) and the Wilcoxon rank-sum test (for continuous variables) were employed.

Patient survival after amputation is shown by Kaplan–Meier curve, and group differences were assessed using a log-rank test. A comparison of survival probabilities at 36 months is based on the log-log transformation described in Klein et al. [11] The Cox proportional hazards regression model assessed group differences via hazard ratios (HR) and 95% confidence intervals (95% CI). A two-sided significance level of 0.05 was adopted for all statistical tests. Statistical analysis was performed using SPSS© statistical software (IBM© SPSS© Statistics, Mac version 28.0.0.0).

## 3. Results

Table 1 shows the clinical characteristics of all patients with PAD admitted to our hospital during the observation period.

**Figure 1:**
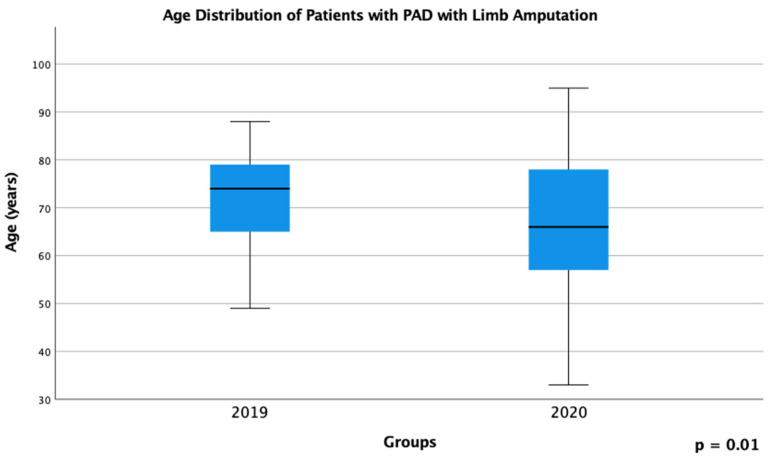
Age distribution of patients with PAD who required limb amputation during the COVID-19 pandemic in 2020 compared to the same period in 2019.

In 2020, patients with PAD who required limb amputation were significantly younger (median age: 66 years) than patients with PAD admitted in 2019 (median age: 74 years; *p* = 0.01; Figure 1).

During the pandemic in 2020, admissions of patients with advanced stage PAD (4–6) requiring limb amputation due to unsuccessful revascularization attempts increased significantly compared to 2019 (*n* = 54 (64%) vs. *n* = 20 (40%); *p* < 0.01; Table 1). On the other hand, it seems that there was a decrease in the proportion of patients admitted with PAD (1–3) during the pandemic in 2020 compared to 2019 (*n* = 30 (36%) vs. *n* = 30 (60%); *p* < 0.01; Table 1). However, it is important to note that these patients required limb amputation due to unsuccessful revascularization. This was largely attributed to the worsening of underlying peripheral atherosclerotic conditions, which were compounded by delays in treatment.

**Figure 2:**
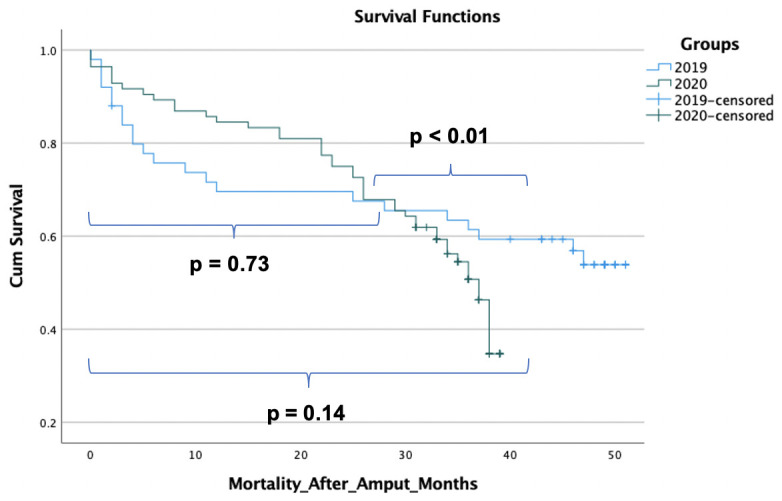
Mortality after limb amputation among patients with PAD treated during the COVID-19 pandemic in 2020 compared to the same period in 2019. *p* = 0.73 represents the mortality difference during the first 26 months; *p* < 0.01 represents the mortality difference after 26 months; *p* = 0.14 represents the mortality differences throughout the whole observational period.

Decreasing survival probabilities were observed among amputees during the COVID-19 pandemic in 2020 throughout the entire observational period. In contrast, mortality among amputees in 2019 decreased after the first 10 months (Figure 2). We did not observe any significant difference in mortality during the first 26 months. However, after 26 months, mortality increased among amputees in 2020 continuously compared to amputees in 2019, where mortality increased slightly (*p* < 0.01).

Table 2 shows the mortality-related characteristics of all admitted amputees with PAD during the observation periods.

The mortality risk was higher in the 2019 cohort for the first year. Thereafter, the mortality risk for amputees in 2020 became higher, leading to a crossing in survival curves after about two years. Thus, the log-rank test was not significant for the observed period. Long-term outcomes after three years showed a mortality probability of 49% in the 2020 cohort and 39% in the 2019 cohort, which are statistically significant (*p* = 0.04). The number of death cases in 2020 was more than double that of 2019 (45 vs. 22), as more amputations were necessary in 2020 and long-term survival in 2020 was worse.

The proportion of major limb amputations among patients with PAD increased significantly in 2020 compared to 2019 (*n* = 55 (65%) vs. *n* = 17 (34%); *p* = 0.03; Table 1). However, we did not observe any significant difference in survival among amputees with major amputation between 2019 and 2020 (*n* = 29 of 55 (53%) vs. *n* = 13 of 17 (77%); *p* = 0.28). We observed significant differences in survival among amputees with major compared to minor amputation (HR = 1.689 (95% CI: 1.026–2.783), *p* = 0.039). Nevertheless, the observation of a high number of deaths among patients who underwent major and minor amputation indicates a detrimental effect of amputation per se on the survival of patients with PAD.

**Figure 3:**
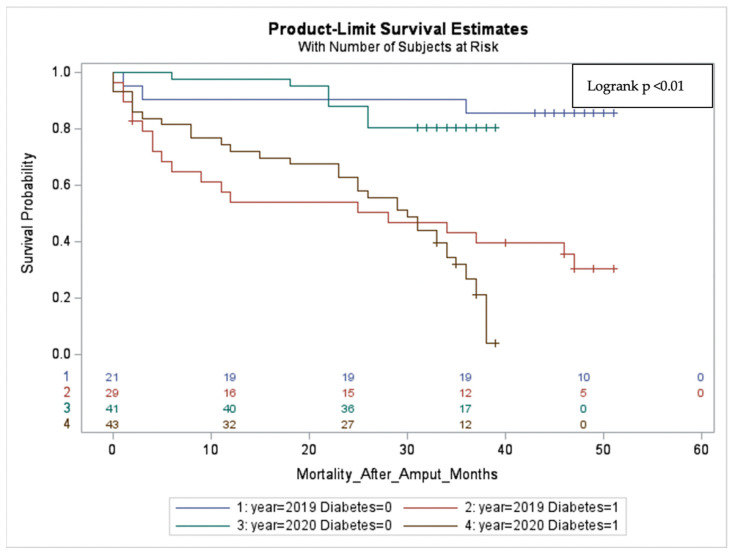
Survival of amputees with PAD with (1) and without (0) diabetes.

The three-year survival of diabetic amputees with PAD in 2020 was worse than that of diabetic amputees in 2019 (27% vs. 50%, respectively; Table 2). A significant difference was observed in the mortality rates of amputees with diabetes compared to those without diabetes over the whole observational period (HR = 6.4; 95% CI: 3.4–12.3; *p* < 0.01). These findings indicate that diabetes is the most significant factor influencing the survival of patients following limb amputation (Figure 3).

A total of 14 of 20 patients with renal insufficiency in 2020 and all 4 patients in 2019 died (HR = 2.2; 95% CI: 1.3–3.7; *p* < 0.01). The presence of diabetes and renal failure and major limb amputation was found to have a significantly negative impact on the survival of patients with PAD following limb amputation.

None of our patients were infected with COVID-19 before admission, during inpatient treatment, or at discharge. Additionally, no COVID-19-related death was documented.

## 4. Discussion

To the best of our knowledge, this is the first study to analyze the short- and mid-term consequences of delayed treatment in patients with PAD following the onset of the COVID-19 pandemic. Our investigation revealed that admissions of patients with advanced-stage PAD (4–6) requiring limb amputation increased significantly during the COVID-19 pandemic in 2020 compared to 2019. Furthermore, the proportion of emergency admissions and major limb amputations among PAD patients increased significantly during the COVID-19 pandemic in 2020 compared to 2019. Although amputees from 2020 were significantly younger than those from 2019, they had a significantly higher long-term mortality risk compared to 2019. According to our observation, twice as many patients died after the COVID-19 pandemic in 2020 compared to the same period in 2019. Moreover, amputees with diabetes and renal insufficiency showed a significantly higher long-term mortality risk compared to amputees without diabetes and renal insufficiency.

Considering an increase in the amputation rate among uninfected patients with PAD who were significantly younger during the pandemic, we observed a post-amputation mortality probability of 49% during the first three years (Table 2). This is still within the lower range of international data, which varies from 40% to 70% [2,3,4].

Several lines of evidence have demonstrated a dramatic shift in cardiovascular mortality from the COVID-19 pandemic in 2020 to the post-pandemic era. Bozzani et al. [12] reported that mortality not related to COVID-19 has doubled in the Lombardy region in Italy since 2020 compared to previous years. Similarly, Woodruff et al. [13] reported an increase in cardiovascular disease mortality in the United States during the 2020 pandemic, which remained high in 2022. Song et al. [14] showed increased mortality rates for younger patients with cardiovascular diseases between 2019 and 2021 in the USA. Interestingly, this increase reversed in 2021–2023. Our observations showed an increased long-term mortality risk among patients with PAD who required limb amputation during the COVID-19 pandemic in 2020, even after three years of follow-up.

Compared to 2019, no significant difference in mortality was observed during the first 26 months. However, after that, mortality increased among amputees in 2020 significantly compared to amputees in 2019, where mortality increased slightly (Figure 2). One potential explanation for this phenomenon is that the amputees from 2020 received delayed treatment due to a lack of self-care. This was likely influenced by the circumstances surrounding the COVID-19 pandemic.

Evidence is emerging that the worst clinical outcomes and high mortality in patients with PAD are associated with lower limb amputation. Remes et al. [15] conducted a study on the impact of major lower limb amputation and multiple comorbidities on mortality in patients with PAD. Their study found that the level of amputation contributed to high mortality rates among those patient populations. Moreover, Kaissar et al. concluded in their meta-analysis that minor amputation also contributes to high mortality in patients with PAD [16]. Nevertheless, in our data, a high number of deaths among patients who underwent major and minor amputation without any significant difference in survival indicates a detrimental effect of amputation on the survival of patients with PAD. However, due to the low number of included cases, further investigations are necessary to address this matter thoroughly.

Thorud et al. [17] indicated renal insufficiency as a risk factor for increased mortality, along with diabetes, in patients with PAD. Additionally, a meta-analysis performed by Stern et al. [18] showed that high mortality rates after limb amputation were mainly linked to comorbidities of PAD, especially diabetes and renal dysfunction. Vrsalovic et al. [19] demonstrated a significant association between diabetes and mortality in patients with advanced-stage PAD. In line with these observations, our data suggest that diabetes and renal insufficiency significantly negatively impact the survival of patients with PAD after limb amputation. Moreover, our findings indicate that diabetes is the most significant factor influencing the survival of patients with PAD following limb amputation (Figure 3).

Along with the retrospective nature of this study, further limitations include the limited scope of the data due to the limited number of patients, which was restricted to one single centre, limited information about the exact cause of death, and a lack of consistent information on the pre-established medication prior to admission.

## 5. Conclusions

Our data confirm the high long-term mortality risk in amputees with PAD, particularly those admitted with advanced stages due to delayed treatment. Comorbidities associated with chronic PAD also contribute to high mortality. Therefore, in future pandemics, continuously monitoring patients with PAD will be crucial to prevent delayed treatment and severe short- and long-term consequences, such as limb amputation and death. This will ensure that these patients do not suffer more from the progression of their chronic condition than from pandemic-related conditions. Therefore, we recommend continuing regular follow-ups while emphasizing the use of appropriate personal protective equipment.

These data provide a focus for further research and improvement, including defining care pathways for patients with PAD, such as telemedicine. These findings might have implications for health policy, clinical care, and patient awareness.

Mohammad Mahdi Kasiri, Martina Mittlboeck, Bernd Gollackner, and Christoph Neumayer.

## Figures and Tables

**Table 1:** The characteristics of patients with PAD.

	2020	2019	*p* Value
No. of admitted patients with PAD	652 (100%)	639 (100%)	
No. of successfully revascularized patients with PAD during the observation periods	568 (87%)	589 (92%)	
Rutherford classification of successfully revascularized patients with PAD			
1–3	454 (80%)	542 (92%)	
4–6	114 (20%)	47 (8%)	
No. of treatments			
Endovascular	98 (15%)	134 (21%)	
Open repair	522 (80%)	358 (56%)	
Conservative	72 (11%)	249 (39%)	
No. of amputees ^1^ after unsuccessful revascularization	84 (100%)	50 (100%)	
Amputation			
Minor ^2^	29 (35%)	33 (66%)	
Major ^2^	55 (65%)	17 (34%)	<0.03
Age of amputees(years)	66 (33–95)	74 (49–88)	0.01
Gender			
Female	27 (32%)	18 (36%)	0.65
Male	57 (68%)	32 (64%)	
Admission			
Elective	23 (27%)	34 (68%)	<0.01
Emergency	61 (73%)	16 (32%)	
Rutherford classification			
1–3	30 (36%)	30 (60%)	<0.01
4–6	54 (64%)	20 (40%)	
Comorbidities			
Treatments			
Endovascular	3 (4%)	1 (2%)	
Open repair	81 (96%)	49 (98%)	
Hypertension	56 (67%)	34 (68%)	0.87
Diabetes	43 (51%)	29 (58%)	0.45
Hyperlipidaemia	50 (60%)	31 (62%)	0.78
Renal insufficiency	20 (24%)	4 (8%)	0.02

^1^ Number of patients with PAD who underwent limb amputation. ^2^ Minor: at the level of the ankle joint or below; Major: above the ankle joint. The median follow-up for all patients was 39 months (95% CI: 34.6–43.4).

**Table 2:** Mortality-related characteristics of all amputees with PAD.

	2020 (*n* = 84)	2019 (*n* = 50)	*p* Value
Dead	45 (54%)	22 (44%)	
Alive	39 (46%)	28 (56%)	
Mortality after 30 days ^3^	3 (4%)	3 (6%)	0.67
All-cause mortality after three years ^3^	49% (CI: 38%–61%)	39% (CI: 27%–54%)	0.04
Median age of dead patients	69 (46–91)	75 (58–86)	0.27
three-year survival of amputees with PAD			
With diabetes	27%	50%	
Without diabetes	80%	86%	

^3^. Number of deceased patients.

## Data Availability

The data that support the findings of this study are available on request from the corresponding author.
